# A noise power spectrum study of a new model‐based iterative reconstruction system: Veo 3.0

**DOI:** 10.1120/jacmp.v17i5.6225

**Published:** 2016-09-08

**Authors:** Guang Li, Xinming Liu, Cristina T. Dodge, Corey T. Jensen, X. John Rong

**Affiliations:** ^1^ Department of Imaging Physics The University of Texas MD Cancer Center Houston TX USA; ^2^ Department of Diagnostic Radiology The University of Texas MD Cancer Center Houston TX USA

**Keywords:** CT, noise power spectrum, model‐based iterative reconstruction, image quality, radiation dose, Veo

## Abstract

The purpose of this study was to evaluate performance of the third generation of model‐based iterative reconstruction (MBIR) system, Veo 3.0, based on noise power spectrum (NPS) analysis with various clinical presets over a wide range of clinically applicable dose levels. A CatPhan 600 surrounded by an oval, fat‐equivalent ring to mimic patient size/shape was scanned 10 times at each of six dose levels on a GE HD 750 scanner. NPS analysis was performed on images reconstructed with various Veo 3.0 preset combinations for comparisons of those images reconstructed using Veo 2.0, filtered back projection (FBP) and adaptive statistical iterative reconstruction (ASiR). The new Target Thickness setting resulted in higher noise in thicker axial images. The new Texture Enhancement function achieved a more isotropic noise behavior with less image artifacts. Veo 3.0 provides additional reconstruction options designed to allow the user choice of balance between spatial resolution and image noise, relative to Veo 2.0. Veo 3.0 provides more user selectable options and in general improved isotropic noise behavior in comparison to Veo 2.0. The overall noise reduction performance of both versions of MBIR was improved in comparison to FBP and ASiR, especially at low‐dose levels.

PACS number(s): 87.57.‐s, 87.57.Q‐, 87.57.C‐, 87.57.nf, 87.57.C‐, 87.57.cm

## I. INTRODUCTION

Computed tomography (CT) is widely used as an essential tool in clinical diagnostic imaging, contributing to about half of medical radiation exposure.^(1)^ Therefore, there are increasing concerns about the radiation exposure to patients from CT.^2^, ^3^ Hence, radiation dose optimization has become of great interest to health‐care providers.^4^, ^5^ Approaches to radiation dose reduction have included improved protocol designs such as eliminating unnecessary acquisitions, improved hardware efficiency, as well as software advancements.

Comparing to filtered back projection (FBP) reconstruction, recent advanced reconstruction algorithms such as iterative reconstruction in CT imaging have shown a dramatic ability to decrease noise while maintaining or improving image quality in the setting of reduced radiation dose. Previously, the major limitation in the implementation of iterative reconstruction in clinical CT imaging was due to intensive computational resources. Initially, a less computationally intensive iterative reconstruction algorithm, adaptive statistical iterative reconstruction (ASiR) was introduced. Due to inherent noise texture in CT images, extremely low‐noise images with unusual noise texture generated by 100% ASiR is not desired by readers. Hence, in practice, a weighted blending of the FBP and ASiR algorithms has been used. Studies show that this approach achieved 32%–65% radiation dose reduction without compromising clinical outcomes.^6^, ^7^, ^8^, ^9^

Computer hardware advancements have recently allowed for reconstruction speeds of purely iterative reconstruction, such as model‐based iterative reconstruction (MBIR), to be acceptable for clinical use, although considerable amount of reconstruction time is still required. For example, depending on applications, the reconstruction time varies from 20 to 80 min for the second generation of GE's Veo (Veo 2.0, GE Healthcare, Waukesha, WI).^(10)^ The second generation of MBIR (e.g., Veo 2.0) has provided significant improvement in clinical imaging. Clinical utilization of MBIR demonstrated further patient radiation dose reduction without necessarily sacrificing diagnostic CT image quality.^11^, ^12^, ^13^, ^14^, ^15^

However, MBIR performs noise reduction differently from FBP and ASiR because of its nonlinear nature. The altered noise texture in images reconstructed by MBIR has been a concern and was of interest for further improvements.^16^, ^17^

The newly released third generation of MBIR (Veo 3.0, GE Healthcare, Waukesha, WI) has become available with reconstruction preset options. Instead of allowing only default “standard” setting in Veo 2.0, Veo 3.0 now provides presets for Texture Enhancement (Texture), Target Thickness (TT), and Recon Setting for various anatomical foci (Table 1). The Recon Setting option includes Standard, Noise Reduction (NR), and Resolution Preference (RP) presets. The presets with prefixes NR and RP are followed by a number representing quantitative performance. For instance, NR05 represents a 5% noise reduction, and RP05 represents a 5% increase in spatial resolution, where the comparison was made between the new options and the Standard setting. This Standard setting is also the only default option in the second‐generation Veo, Veo 2.0. The new Texture Enhancement option of Veo 3.0, which is not included in Veo 2.0, rebalances the noise distribution to achieve a more isotropic noise behavior in 3D to reduce undesired image distortion and streak artifacts,^(16)^ which aims to improve overall image appearance.

Noise power spectrum (NPS) analysis has been a powerful tool in characterizing noise properties in medical images. Specifically, we conducted 2D NPS analysis for a better understanding of reconstruction algorithm performance in noise reduction. Although some studies have been performed by other researchers to investigate NPS on Veo 2.0,^18^, ^19^, ^20^, ^21^ in‐depth NPS studies are needed to understand the new reconstruction presets in Veo 3.0. In this study, we performed NPS analysis on CT phantom images reconstructed with FBP, ASiR, Veo 2.0, and Veo 3.0. The images were acquired using clinical imaging parameters on a clinical CT scanner. The phantom mimics patient size and shape.

**Table 1:** Comparison of Reconstruction Settings in Veo 3.0 and Veo 2.0.

	*Veo 3.0*	*Veo 2.0*
Texture Enhancement	On / Off	N/A
Target Thickness	0.625, 1.25, 2.5, 3.75 or 5.0 mm	0.625 mm
Recon Setting^a^	Abd/Pelvis : NR05, RP05, Standard	Standard
	Head/Neck : NR40, Standard	
	Thorac : RP05, RP20	

^a^ Five reconstruction settings are available: NP05, NP40, RP05, RP20, and Standard. NR05 and NR40 represent 5% and 40% noise reduction, respectively, in comparison with the standard setting. RP05 and RP20 represent 5% and 20% resolution increase, respectively, in comparison with the standard setting. Each of the five settings is available only to certain anatomical focuses (e.g., NR40 is only available to Head/Neck protocols).

## II. MATERIALS AND METHODS

**Figure 1:**
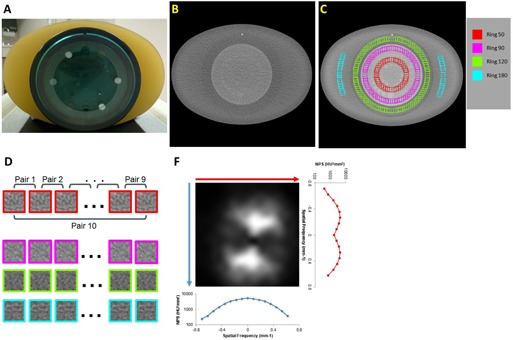
Catphan 600 CTP515 module, surrounded by CTP579 uniformity body annulus, an oval, fat‐equivalent ring, mimicking a patient abdomen (a) was scanned on a GE HD750 CT scanner. The axial images (b) were sampled using four ROI groups as shown in four colors (c), located along four concentric rings with radii of 50, 90, 120, and 180 pixels from the center of the phantom. Six ${\text{CTDI}}_{\text{vol}}$ levels were used for this study. Ten scans were conducted for each dose level. The noise was obtained by subtracting the same ROI in two consecutive images (d). The mean 2D NPS of the ROIs on the same ring was calculated (f). Then, the 1D mean NPS along the horizontal direction were labeled as NPS Horizontal and the one along the vertical direction were labeled as NPS Vertical.

The Catphan 600 CTP515 module (The Phantom Laboratory, Salem, NY), surrounded by an oval, fat‐equivalent ring to mimic patient size/shape (Fig. 1), was scanned on a GE HD750 CT scanner at six ${\text{CTDI}}_{\text{vol}}$ levels: 0.97, 1.69, 2.9, 5.81, 11.62, and 18.88 mGy with typical parameters for abdomen scans: 120 kVp, 0.8 s rotation time, beam width of 40 mm (0.625mm×64), a pitch of 0.984, large scan field of view (SFOV) and 36 cm display field of view (DFOV). The ${\text{CTDI}}_{\text{vol}}$ levels were selected to cover the typical clinical dose levels (e.g., 18.88 and 11.62 mGy), as well as low‐ and ultra‐low‐dose levels (e.g., 0.97 mGy) for abdomen protocols. At each ${\text{CTDI}}_{\text{vol}}$ level, 10 repeated scans were acquired to achieve sufficient data sampling, as well as nonzero mean (DC components) removal and signal detrending through the subtraction of consecutive scans. Then 2.5 mm images were reconstructed using standard algorithm with FBP; 20%, 40%, and 70% ASiR; Veo 2.0 and 3.0 with various combinations of new presets.

Veo 3.0 was installed on another identical GE HD750 CT scanner. Therefore, the raw data acquired with the first HD750 were able to be uploaded onto this second one and then be reconstructed using the Veo 3.0 retro reconstruction. The raw data were first reconstructed with a slice thickness of 0.625 mm, which were then reformatted with a slice thickness of 2.5 mm regardless of the Target Thickness being 0.625 or 2.5 mm. Only one image from the center of the image stack of each scan was used for the NPS analysis. Hence, there were a total of 10 images being extracted for the NPS analysis for each combination of dose levels and reconstruction algorithms or, in the case of Veo 3.0, reconstruction presets.

To evaluate the 2D spatial NPS variation with respect to the distance to the center of DFOV, four ROI groups were placed on four concentric circles centering at the center of the phantom. The radii of the circles were 50, 90, 120, and 180 pixels. Each of these ROI groups was sampled along one of the four concentric circles in the images (Fig. 1). The size of the ROIs was 16×16 pixels and their centers were placed on these four circles, which essentially formed four concentric sample rings. Two adjacent ROIs on the same circle had a center‐to‐center distance of 8 pixels on the circumference. The noise of the j‐th ROI on the circle $C_{k}(k=1,2,3$, and 4) in the i‐th image (i=1,2,…,10) was:

(1) $$I_{\text{noise}}(i,j,C_{k})=\left(ROI_{i,j,C_{k}}–ROI_{i+1,j,C_{k}}\times \frac{\text{mean}(ROI_{i,j,C_{k}})}{\text{mean}(ROI_{i+1,j,C_{k}})}\right)\times \frac{1}{\sqrt{2}}$$

where $ROI_{i+1,j,Ck}=ROI_{1,j,Ck}$ when i=10. The NPS was then calculated for each $I_{\text{noise}}(i,j,C_{k})$ and the NPS of the circle $C_{k}$ in the i‐th image was:

(2) $$NPS(f_{x},f_{y},i,C_{k})=\frac{\sum_{j}{\left\{DFT_{2D}[I_{\text{noise}}(i,j,C_{k})]\right\}}^{2}}{\text{num}\;of\;ROIs}\times \frac{\Delta x\Delta y}{N_{x}\times N_{y}}$$

The 1D mean of NPS along the horizontal and vertical direction of this ROI group were

(3) $$NPS_{\text{vertical}}(C_{k})=\frac{1}{10}\frac{1}{N_{x}}\sum_{i=1,\cdots ,10}\sum_{f_{x}}NPS(f_{x},f_{y},i,C_{k})$$

(4) $$NPS_{\text{horizontal}}(C_{k})=\frac{1}{10}\frac{1}{N_{y}}\sum_{i=1,\cdots ,10}\sum_{f_{y}}NPS(f_{x},f_{y},i,C_{k})$$

**Figure 2:**
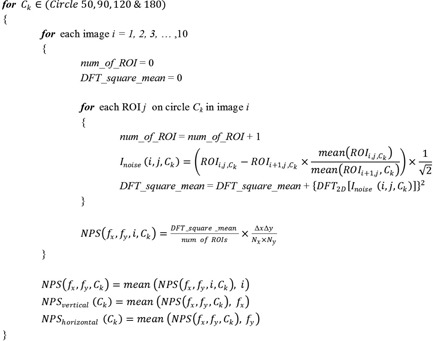
Pseudocode for NPS calculation.

A pseudocode (Fig. 2) is also provided to delineate the entire NPS calculation process.

For each reconstruction algorithm at each dose level, the 1D‐mean NPS curves of each of the four ROI groups (four sample rings) were calculated. Comparisons were conducted 1) among four ROI groups at the same dose level with the Veo 3.0 and FBP reconstruction algorithms; 2) between Target Thickness of 0.625 and 2.5 mm; 3) between reconstructions with and without Texture Enhancement; 4) between Veo 3.0 and 2.0; 5) among all dose levels with the Veo 3.0 or FBP reconstruction algorithms at the radius of 120 pixels; and 6) among all aforementioned reconstruction algorithms at each of the investigated dose level at the radius of 120 pixels.

## III. RESULTS

**Figure 3:**
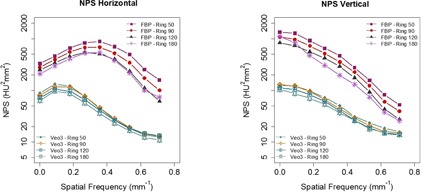
Plots of ${\text{NPS}}_{\text{horizontal}}$ and ${\text{NPS}}_{\text{vertical}}$ at ${\text{CTDI}}_{\text{vol}}$ of 5.81 mGy with Veo 3.0 (TT 2.5 mm+NR05+Texture) and FBP. In addition to their differences in ${\text{NPS}}_{\text{horizontal}}$ peak frequencies, the noises with Veo 3.0 had lower magnitude and distributed in a narrower range, in comparison to the FBP. In general, the noises increased when ROIs moved towards the center. There exists the exception of noise on the most outer ring with a 180‐pixel radius being higher at some frequencies than the ones on the inner rings. These discrepancies may be caused by 1) match of the phantom shape (elliptical section instead of circular) and size with the bowtie filter, 2) types of materials in the samples, and 3) anisotropic sampling: this ring samples only the lateral regions of a circle, instead of a complete circle. However, further investigation may be needed to understand the discrepancies.

At ${\text{CTDI}}_{\text{vol}}$ of 5.81 mGy, the NPS of Veo 3.0 with TT2.5mm+Texture+NR05 (Fig. 3) had lower magnitude at all frequencies and were distributed in a much narrower range compared to the NPS of FBP, indicating more spatially homogeneous noise. Veo 3.0 and FBP had different ${\text{NPS}}_{\text{horizontal}}$ peak frequencies (0.1/mm vs. 0.3 ~ 0.4/mm), indicating there were differences in their noise texture. Also, in general, the noises increased as the ROIs moving towards the center of the image. The exceptions occurred on the most outer ring (with a radius of 180 pixels), where the noise power at some frequencies were higher than those on the inner rings.

**Figure 4:**
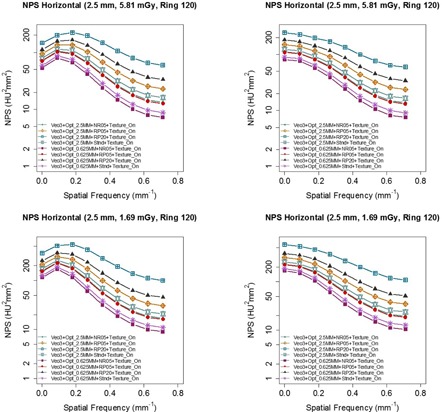
NPS of images with ${\text{CTDI}}_{\text{vol}}$ of 5.81 mGy reconstructed with various combinations of Veo 3.0's new reconstruction presets, indicating Target Thickness of 0.625 mm had better noise reduction than Target Thickness of 2.5 mm. The Texture Enhancement option was turned on for all the images. The options included for this comparison were between Target Thickness of 2.5 mm vs. 0.625 mm with recon options of RP20, RP05, NR05, and Stnd. The images with Target Thickness of 0.625 mm were first reconstructed into 0.625 mm images and they were then reformatted into 2.5 mm images for NPS analysis.

The Target Thickness setting of 0.625 mm generated images with less noise than the setting of 2.5 mm, when the other options remained the same (Fig. 4). The noise in reconstructed images decreased in the order of: RP20, RP05, Standard, and NR05, when the other options remained the same.

**Figure 5:**
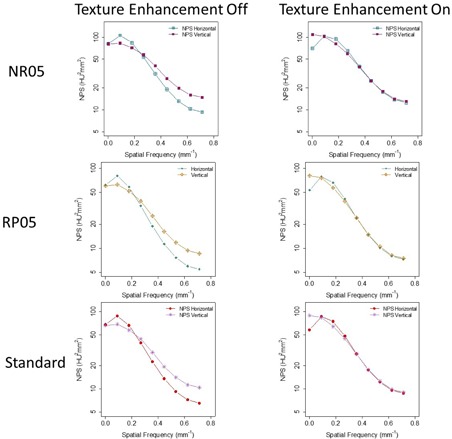
Comparison between images reconstructed with (left column) and without (right column) Texture Enhancement for ${\text{CTDI}}_{\text{vol}}$ levels of 5.81 mGy. NPS analyses indicated that ${\text{NPS}}_{\text{horizontal}}$ and ${\text{NPS}}_{\text{vertical}}$ were better matched when Texture Enhancement was applied, indicating more isotropic noise behavior. Target Thickness was set to 0.625 mm for the maximum noise reduction. The images were first reconstructed into 0.625 mm images and they were then reformatted into 2.5 mm images for NPS analysis.

**Figure 6:**
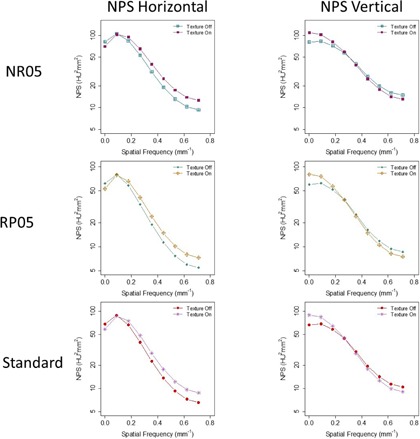
Same data from Fig. 5 organized by the NPS dimensions, emphasizing how the Texture Enhancement feature altered noise power to achieve isotropic noise behavior.

**Figure 7:**
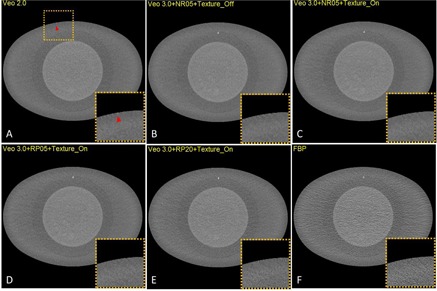
Jagged edges in VEO 2.0 (a) at ${\text{CTDI}}_{\text{vol}}$ of 5.81 mGy, in comparison with the same image reconstructed with Veo3+ TT0.625 mm+NR05+Texture (b), Veo3+TT0.625 mm+NR05+ (c), Veo3+TT 0.625 mm+RP05+Texture (d), Veo3+TT 0.625 mm+RP20+Texture (e), and FBP (f). The Texture Enhancement indeed reduced the artifacts.

Figure 5 shows that ${\text{NPS}}_{\text{horizontal}}$ and ${\text{NPS}}_{\text{vertical}}$ were better matched when Texture Enhancement was turned on, suggesting that Texture Enhancement achieved its design goal of more isotropic noise behavior. Figure 6 shows how the Texture Enhancement altered noise power at all frequencies to achieve this goal. In addition, this option reduced artifacts. Figure 7 shows that jagged edge between air and the phantom in the Veo 2.0 image was reduced when Texture Enhancement option was turned on. However, compared to the FBP, this artifact was still visible in the Veo 3.0 image.

**Figure 8:**
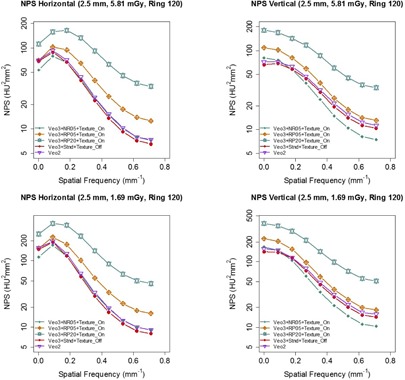
Comparison between Veo 2.0 and 3.0. Target Thickness was set to 0.625 mm for Veo 3.0. These Veo 3.0 images were reconstructed with 1) NR05+Texture, 2) PR05+Texture, 3) PR20+Texture, and 4) Standard only. The images were first reconstructed into 0.625 mm images and they were then reformatted into 2.5 mm images for NPS analysis. NPS of Veo 2.0 was very similar to that of Veo 3.0 with TT0.625 mm+Standard.

Figure 8 suggests that Veo 2.0 and Veo 3.0 were very similar with respect to their NPS when Veo 3.0 used Target Thickness of 0.625 mm and Standard reconstruction option without Texture Enhancement. This suggests Veo 3.0 can be configured to behave similarly to Veo 2.0 with respect to noise. Also, Veo 3.0 with Target Thickness of 0.625 mm and NR05 recon setting generated images with slightly less noise compared to Veo 2.0, suggesting further noise reduction is feasible although some other image quality metrics of the reconstructed images are expected to be affected.

**Figure 9:**
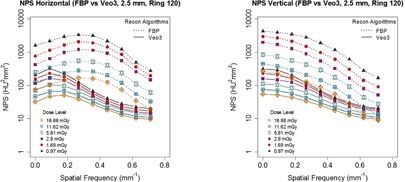
${\text{NPS}}_{\text{horizontal}}$ and ${\text{NPS}}_{\text{vertical}}$ with Veo 3.0 (TT 2.5 mm+NR05+Texture) and FBP at all six dose levels (Ring 120). Veo 3.0 ${\text{NPS}}_{\text{horizontal}}$ peaks appeared to shift from 0.2/mm to 0.1/mm when the ${\text{CTDI}}_{\text{vol}}$ decreased from 19 mGy to 1 mGy, indicating Veo 3.0 noise reduction is dose dependent. Also, compared with FBP, Veo 3.0 images had much lower noise, especially at low‐dose levels.

As expected, higher dose level resulted in lower noise (Fig. 9). This trend was observed in every ROI group (only Ring 120 is shown) and with every reconstruction algorithm (only FBP and Veo 3.0 are shown). The figure also showed that Veo 3.0's ${\text{NPS}}_{\text{horizontal}}$ peak shifted from 0.2/mm to 0.1/mm when the ${\text{CTDI}}_{\text{vol}}$ decreased from 19 mGy to 1 mGy, further supporting the noise reduction of Veo 3.0 was dose/noise dependent, which was also seen in Veo 2.0^(18)^.

**Figure 10:**
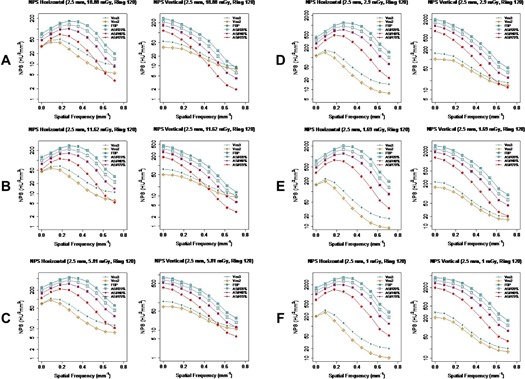
Comparison of ${\text{NPS}}_{\text{horizontal}}$ and ${\text{NPS}}_{\text{vertical}}$ of various reconstruction algorithms at all six ${\text{CTDI}}_{\text{vol}}$ levels. Veo 3.0 images were reconstructed with TT 2.5mm+NR05+Texture. NPS of Veo 2.0 and 3.0 had overlap with the others at higher frequency at higher dose levels, however they were completely separated at low‐dose level (${\text{CTDI}}_{\text{vol}}<3\text{mGy}$).

The comparison among all the reconstruction algorithms at various dose levels (Fig. 10) showed that Veo 2.0 and a typical Veo 3.0 setting (TT 2.5 mm+NR05+Texture) overall had lower ${\text{NPS}}_{\text{horizontal}}$ and ${\text{NPS}}_{\text{vertical}}$ magnitudes at all dose levels at low and intermediate frequencies than the others, although the differences were small at typical clinical dose levels (i.e., 18.88 and 11.62 mGy). At higher spatial frequency (>0.5/mm), on the other hand, there were small overlaps between NPS of the two generations of Veo reconstructions and the others. The overlaps started diminishing at ${\text{CTDI}}_{\text{vol}}$ levels around 3 mGy. These findings suggest that these MBIR algorithms completely outperformed ASiR and FBP only at low‐ and ultra‐low‐dose levels.

## IV. DISCUSSION

The observation of the peaks of ${\text{NPS}}_{\text{horizontal}}$ and ${\text{NPS}}_{\text{vertical}}$ occurring at different frequencies was consistent for all reconstruction algorithms at all dose levels. This observation was different from radially symmetrical 2D NPS reported by other studies.^18^, ^22^, ^23^, ^24^ However, those radially symmetrical 2D NPS images were found with small phantoms having circular sections, in contrast to our phantom with elliptical shape. In a sinogram, the noise of projections is angle dependent because the angle‐dependent varying thickness of the phantom results in an anisotropic noise behavior with fixed tube current. When an elliptical object was used in an analytical and simulated study,^(25)^ a 2D NPS image similar to ours was also reported.

The noise is inversely correlated with distance to the reconstruction center since the X‐ray fluence transmitted to the detector varied spatially.^25^, ^26^, ^27^ Our results are in agreement with these findings in general, although noise in Veo 3.0 images were more spatially homogeneous in comparison to FBP.^(18)^ However, there exists the exception of noise on the most outer ring with a 180‐pixel radius being higher than the ones on the inner rings at some frequencies (Fig. 3). These discrepancies may be caused by the differences between ROIs on the most outer ring and the others in 1) match of the phantom shape (elliptical section instead of circular) and size with the bowtie filter, 2) types of materials in the ROI samples: this ring locates on the CTP579 uniformity body annulus while the others on the Catphan 600 CTP515 module, and 3) anisotropic sampling: this ring samples only the lateral regions of a circle, instead of a complete circle. However, further investigation may be needed to understand the discrepancies.

A thicker Target Thickness setting generated images with more noise (Fig. 4). To maintain a certain noise level in the final reconstructed images, noise needed to be further reduced in thinner images than in thicker images (0.625 mm vs. 2.5 mm). However, when these thinner images (0.625 mm) were combined to form a thicker image (2.5 mm), the noise appeared to be lower in these newly combined thicker images than in the images directly reconstructed with the Target Thickness setting of the same thickness (2.5 mm) by Veo 3.0. It seems that this Target Thickness setting is designed to optimize the use of computational resource for varying needs of noise reduction. After all, a thicker image slice needs less noise reduction to achieve the same noise level as does a thinner image slice. Texture Enhancement is designed to achieve a more isotropic noise behavior and artifact reduction, which is supported by our findings (Fig. 5 and Fig. 7). Reconstruction settings like NR05, RP05, and RP20, in addition to the default “standard” setting, give Veo 3.0 flexibility to better satisfy different needs for different anatomical foci, although they altered NPS at the same time.

Veo 3.0 and 2.0, in general, achieved better noise reduction than ASiR and FBP. However, at typical clinical dose levels, the outcome differences among Veo, ASiR, and FBP were small; while at higher frequencies (<0.5/mm), ASiR and FBP even produced images with lower noise. Only when the dose level was further reduced (${\text{CTDI}}_{\text{vol}}<3\;\text{mGy}$), both generations of Veo reconstruction outperformed the others at all frequencies, in term of NPS. This finding suggests that the Veo reconstructions perform better at low‐ and ultra‐low‐dose levels for which they were intended and they are less beneficial at typical dose levels.

The results of our study help us to better understand the advanced reconstruction options for achieving patient dose reduction while maintaining acceptable diagnostic image quality. Future perceptional reader study based on clinical patient images will ultimately reveal the effectiveness of Veo 3.0 reconstruction.

## V. CONCLUSIONS

The third‐generation MBIR (Veo 3.0) provides options to better satisfy clinical needs. Depending on clinical applications, Veo 3.0 can be configured to behave similarly to Veo 2.0 or to achieve further noise reduction. As designed for achieving patient dose reduction, the overall noise reduction performance of both generations of MBIR was improved compared to the other reconstruction algorithms such as FBP and ASiR, especially at very low‐dose levels. Proper reconstruction setting choices and configuration are critical to achieve optimized clinical CT imaging.

## ACKNOWLEDGEMENT

This work was performed in collaboration with General Electric Healthcare Technologies at the Center for Advanced Biomedical Imaging, University of Texas MD Anderson Cancer Center.

## COPYRIGHT

This work is licensed under a Creative Commons Attribution 3.0 Unported License.
